# Use of Insecticide-Treated House Screens to Reduce Infestations of Dengue Virus Vectors, Mexico

**DOI:** 10.3201/eid2102.140533

**Published:** 2015-02

**Authors:** Pablo Manrique-Saide, Azael Che-Mendoza, Mario Barrera-Perez, Guillermo Guillermo-May, Josue Herrera-Bojorquez, Felipe Dzul-Manzanilla, Cipriano Gutierrez-Castro, Audrey Lenhart, Gonzalo Vazquez-Prokopec, Johannes Sommerfeld, Philip J. McCall, Axel Kroeger, Juan I. Arredondo-Jimenez

**Affiliations:** Universidad Autónoma de Yucatan, Merida, Mexico (P. Manrique-Saide, M. Barrera-Perez, G. Guillermo-May, J. Herrera-Bojorquez);; Servicios de Salud de Yucatán, Gobierno del Estado de Yucatan, Merida (A. Che-Mendoza);; Servicios Estatales de Salud de Guerrero, Chilpancingo, Mexico (F. Dzul-Manzanilla, C. Gutierrez-Castro);; Centers for Disease Control and Prevention, Atlanta, Georgia, USA (A. Lenhart);; Emory University, Atlanta (G. Vazquez-Prokopec);; World Health Organization, Geneva, Switzerland (J. Sommerfeld, A. Kroeger);; Liverpool School of Tropical Medicine, Liverpool, UK (P.J. McCall);; Centro Nacional de Programas Preventivos y Control de Enfermedades, Mexico City, Mexico (J.I. Arredondo-Jimenez)

**Keywords:** viruses, dengue, arbovirus, mosquitoes, Aedes aegypti, vector, control, prevention, outbreak, epidemic, net, LLIS, vector-borne infections, Mexico, screens, insecticide, *Suggested citation for this article*: Manrique-Saide P, Che-Mendoza A, Barrera-Perez M, Guillermo-May G, Herrera-Bojorquez J, Dzul-Manzanilla F, et al. Use of insecticide-treated house screens to reduce infestations of dengue virus vectors, Mexico. Emerg Infect Dis [Internet]. 2015 Feb [*date cited*]. http://dx.doi.org/10.3201/eid2102.140533

## Abstract

Dengue prevention efforts rely on control of virus vectors. We investigated use of insecticide-treated screens permanently affixed to windows and doors in Mexico and found that the screens significantly reduced infestations of *Aedes aegypti* mosquitoes in treated houses. Our findings demonstrate the value of this method for dengue virus vector control.

Vector control is the primary method for prevention and control of the increasingly frequent dengue outbreaks that threaten more than half the global human population (*1*). Existing approaches target breeding sites or attack adult mosquitoes by insecticide space-spraying, but these methods, at best, offer only immediate solutions and are rarely effective or sustainable for the long term (*2*). Methods that target the largely endophilic adult female *Aedes aegypti* mosquito vectors within buildings where they rest and bloodfeed have greater potential for sustained results and acceptance at the community level. One such method, long-lasting insecticidal-net (LLIN) curtains hung at windows or doors, can greatly reduce vector populations at high coverage rates (*3**–**5*), but efforts are compromised when curtains remain open during daytime or when all house entry points cannot be protected (*6**,**7*). Fixed or permanent screens covering doors and windows could eliminate this problem. Mosquito-proofing of houses is effective in malaria control (*8*), and reduced risk for dengue has been associated with the use of untreated (*9*) and insecticide-treated (*3**,**10*) screens.

## The Study

**Figure 1:**
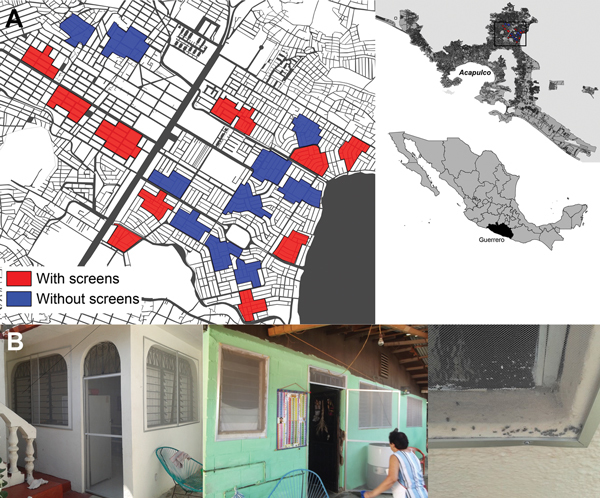
Area of study of long-lasting insecticide-treated screens in Acapulco, Mexico, March 2011–March 2013. A) Locations of clusters in the neighborhoods of Ciudad Renacimiento and Zapata, showing areas with (red) and without (blue) screens. Insets show location of study area (black box) in Acapulco and Guerrero state (black shading) in Mexico. B) Photographs of screens mounted on aluminum frames and fixed to windows and external doors of treated houses in 2012. The insects visible in the right photograph are dead house flies.

During 2011–2013, in the city of Acapulco in Guerrero state, Mexico (Figure 1), an area of consistently high dengue transmission (http://www.epidemiologia.salud.gob.mx/dgae/panodengue/intd_dengue.html), we investigated the effect on vector infestations of permanently mounted, insecticide-treated screens fitted to doors and windows of residential houses. The screens (Duranet, Clarke Mosquito Control, Roselle, IL, USA) were made of 0.55% wt/wt α-cypermethrin–treated nonflammable polyethylene netting (145 denier; mesh = 132 holes/in^2^); the design is approved by the World Health Organization (WHO) Pesticide Evaluation Scheme (http://www.who.int/whopes/en/).

We used a cluster-randomized sampling design constructed on the basis of earlier studies (*4**–**6**,**11*) to select 20 clusters (10 treatment, 10 control; 100 households/cluster) from a possible 30 clusters by using digital maps (Google Earth software; Google Inc., Mountain View, CA, USA) (Figure 1). Sample size was determined by using a 2-level hierarchical model to achieve 80% power at a 5% level of significance. Thus, for a negative binomial distribution with a dispersion coefficient of 0.02 and intracluster coefficient of 0.05, a minimum of 8.9 clusters/arm were required. Written informed consent was obtained from participating households; the WHO Ethical Review Committee (WHO reference no. 2010/82951-0, unit reference no. A90297) and Guerrero State Ministry of Health granted ethical permission for the study.

Participating households in the treatment arm were instructed on LLIS maintenance during installation (April–December 2012). Control houses received no treatment. Five entomologic surveys of randomly selected houses were conducted: before intervention (March 2011, September 2011, March 2012) and at 5 and 12 months after intervention (September 2012, March 2013; wet and dry seasons, respectively). Before intervention, 32 houses per cluster were sampled at each survey; after intervention, 210 houses from treated clusters and 302 from control clusters were sampled in September 2012 and 311 houses from treated and 320 from control clusters in March 2013.

Indoor resting adult mosquitoes were collected by using modified CDC backpack aspirators (John W. Hock Co., Gainesville, FL, USA) from all houses in a cluster on the same day during 9 am–3 pm. Indices for *Ae. aegypti* mosquitoes (the only *Aedes* species found) were calculated to quantify house infestation (percent of all houses positive) and infestation density (numbers per infested house) for all mosquitoes, all females, all blood-fed females, and males.

For presence–absence data, we performed logistic regression models with a single predictor variable identifying houses with LLIS and control houses (coded as 1 and 0, respectively) and accounting for each house membership in a given sampling cluster (cluster-robust SE calculation). Odds ratios (ORs) and 95% CIs indicating the effect of LLIS on each entomologic indicator were calculated. Overdispersed index data were compared between arms by using the Mann-Whitney U test. The effect of treatment on each metric was analyzed by negative-binomial regression using, as with the logistic models, treatment as the sole predictor variable (1 and 0 coding). Negative binomial models also accounted for membership of a house in a sampling cluster (cluster-robust SE calculation). ORs and incidence rate ratios (IRRs) were calculated with 95% CIs; significance was set at p<0.05. Analyses were performed by using Stata 12.0 (StataCorp, College Station, TX, USA).

**Figure 2:**
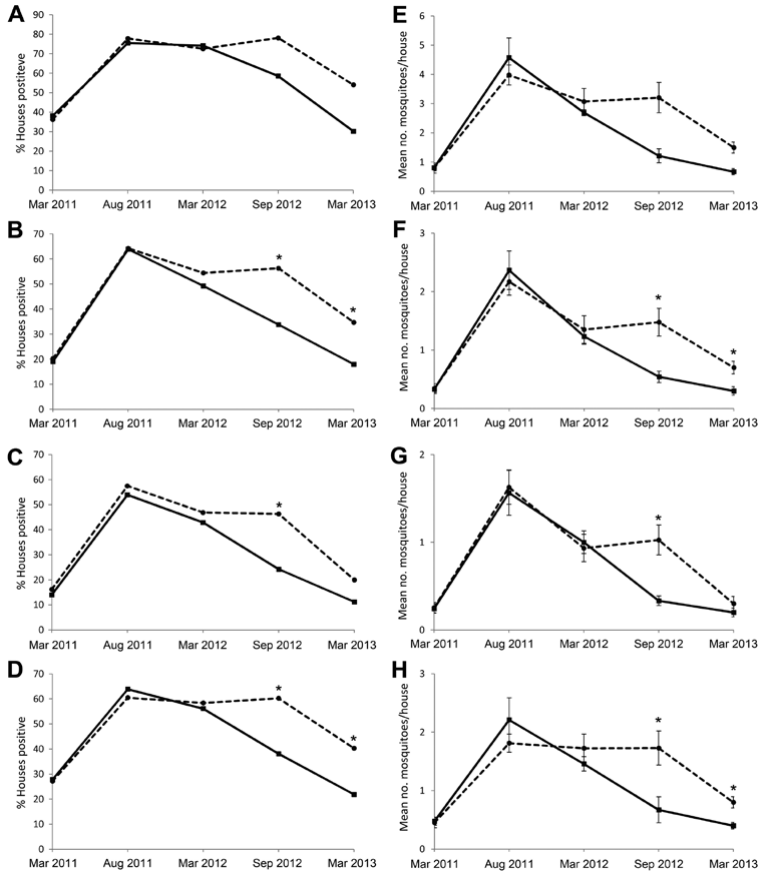
Infestation indices for adult *Aedes aegypti* mosquitoes in intervention (solid lines) and control (dashed lines) households before and after intervention in Acapulco, Mexico, as measured during dry (March) and wet (August–September) season cross-sectional surveys, 2011–2013. A–D) Vector prevalence: percentage of houses positive for A) all adults; B) all females; C) blood-fed females; D) males. E–H) Vector density: mean number per infested house for E) all adults; F) all females; G) blood-fed females; H) males. Error bars indicate SEs. Fitting of insecticide-treated window and door screens commenced during April 2012. Asterisks (*) denote dates when the index was significantly different between treated and control groups.

Before intervention, indices were similar for both study arms on all sampling dates. House infestation rates (Figure 2, panels A–D) and mosquito densities (Figure 2, panels E–H) followed seasonal patterns (2-sample Wilcoxon rank-sum test for all treatment–control comparisons, |z|<1.0; p>0.1). At 5 months postintervention, significantly fewer treated than control houses were infested with *Ae. aegypti* adult female mosquitoes (OR 0.38, 95% CI 0.21–0.69), blood-fed females (OR 0.36, 95% CI 0.21–0.60), and males (OR 0.39, 95% CI 0.19–0.77). A significant effect was still seen at 12 months for adult females (OR 0.41, 95% CI 0.25–0.68) and males (OR 0.41, 95% CI 0.27–0.64) but not for blood-fed females (OR 0.51, 95% CI 0.24–1.05). Analyses of infestation density showed similar trends, with significantly fewer *Ae. aegypti* mosquitoes found in treated than in control houses: adult females at 5 (IRR 0.37, 95% CI 0.27–0.49) and 12 (IRR 0.40, 95% CI 0.23–0.70) months postintervention, males at 5 (IRR 0.39, 95% CI 0.28–0.54) and 12 (IRR = 0.49, 95%CI 0.33–0.72) months postintervention, and blood-fed females at 5 (IRR 0.32, 95% CI 0.23–0.45) but not 12 (IRR 0.49, 95% CI 0.23–1.05) months postintervention.

A comparison of wet season data from treatment houses before (August 2011) and after (September 2012) intervention showed that significantly fewer females and blood-fed females were found postintervention (Wilcoxon matched pairs W = 30706, *z* = 3.717, and W = 20706, *z* = 3.146; p<0.05 for both comparisons). However, the number of male mosquitoes did not change significantly (W = 20706, *z* = 1.385; p>0.05).

At 5 months postintervention, fewer LLIS-treated houses (33%) than control houses (56%) remained infested with female *Ae. aegypti* mosquitoes. Lower numbers of female mosquitoes were also found per infested house (0.54 ± 0.9) than per control house (1.39 ± 2.0); this effect was still detectable at 12 months postintervention (18%, 0.3 ± 0.8, vs. 35%, 0.7 ± 1.4).

## Conclusions

In our study, the entomologic effect of LLIS was greater than that detected in a recent study of deltamethrin-treated window curtains (*12*), in which a 27% reduction of adult *Ae. aegypti* mosquitoes was only sustained for a short time after curtain installation. Other studies of insecticide-treated curtains in Latin America have reported entomologic effects by using immature stage indicators alone (*4*,*5*,*7*). Whether these reductions were sufficient to affect dengue transmission is unknown, and the overall effect on dengue infections remains to be evaluated.

Our results are encouraging in view of high levels of insecticide resistance in *Ae. aegypti* mosquitoes in Acapulco. Although resistance to α-cypermethrin has yet to be reported in Guerrero, high frequencies of mutations in the voltage-gated sodium channel gene, which is associated with pyrethroid resistance in *Ae. aegypti* mosquitoes, have been reported (*13*). If insecticide resistance began to reduce the efficacy of the method we describe, the screens could be treated with different insecticide classes. 

We found the use of LLIS was a popular intervention, and perceived efficacy was reinforced by a reduction in other domestic pests (Figure 1) (*14*)*.* The likely effects on other peridomestic disease vectors could promote increased adoption of the intervention with additional cost benefits. The polyethylene netting was durable on windows; it was often damaged on the lower sections of doors (*14*) but readily repaired by reinforcement with metal mesh.

Dengue vector control programs using house screens are ongoing in selected cities in Mexico and Brazil. These results were obtained during an exploratory phase of that initiative. Stakeholders in other countries may also consider evaluating this novel approach for dengue vector control.
